# Supplementary material for: Designing a hybrid dimension reduction for improving the performance of Amharic news document classification
Source: PLoS One. 2021 May 21;16(5):e0251902. doi: 10.1371/journal.pone.0251902 (PMC8139506; doi:10.1371/journal.pone.0251902)
Supplement: Attachment — Submitted filename: Response to Reviewers.docx [file pone.0251902.s001.docx]

**Rebuttal Letter**

Dear Thippa Reddy Gadekallu,

We were pleased to have an opportunity to revise our manuscript now entitled “Designing a Hybrid Dimension Reduction for Improving the Performance of Amharic News Document Classification ” (PONE-D-21-09681). In revised manuscript, we have carefully considered reviewers’ comments and suggestions. As instructed, we have attempted to succinctly explain changes made in reaction to all comments. We reply to each comment in point-by-point fashion. We have color coded revised manuscript as text. The responses to the concerns raised by reviewers are below and are color coded as follows: a) Comments from editors or reviewers are shown as *text* in italic font b) Our responses are shown as text.

The reviewers’ comments were very helpful overall, and we are appreciative of such constructive feedback on our original submission. After addressing the issues raised, we feel the quality of the paper is much improved.

Sincerely

Demeke Endalie

**Reviewer 1**

1. *Contributions and novelty are major aspects of a research work to be accepted; I find those lacking*

A: We appreciate your insightful suggestions. We agree that it is better to provide contribution of the study. We discussed them at the end of the introduction by paragraph and bullet.

2. *Come up with strong literature review, presentation of the algorithms, mathematical model and rigorous analysis of the results.*

A: Thank you very much. We revise the relevant works and compare our method to new dimension reduction methods. In addition, in the methodology section, we described our algorithm. The section on results and discussion is strengthened.

3. *Reference*

A: We appreciate your insightful suggestions. We presented related papers that was published in 2020 and 2021.

**Reviewer 2**

1. *Please improve the abstract*

A: We appreciate your insightful suggestions. We have corrected it in the abstract section of revision.

2. *Introduction is too long. Please reduce it. It should be specific.*

A: Thank you very much. We've rewritten it.

3. *Improve the contribution list at the end of the introduction and organization paragraph.*

A: We appreciate your insightful suggestions. We agree that it is better to provide contribution of the study. We discussed them at the end of the introduction by paragraph and bullet.

4. *Result section is written badly. Explain results extensively and clearly explain the flow of results.*

A: Thank you very much. We've rewritten it in the result and discussion section of revision.

5. *Explain the dataset in detail and add references. What is the motivation behind selecting this dataset?*

A: We appreciate your insightful suggestions. We have provided the motivation as well as the link to obtain it.

6. *Abbreviations and Acronyms should be added only first time. Then only acronyms should be used in the entire paper*

A: Thank you very much. We have reviewed it.

7. *Few latest references can be added in the related work.*

A: We appreciate your insightful suggestions. We include the most recent related works.

8. *Problem statement must be in the introduction.*

A: Thank you very much. When we updated the introduction section, we revised it.

9. *Grammar check is required.*

A: Thank you very much. We were able to get away with it thanks to linguistics.

**Reviewer 3**

1. *The contributions of the authors are not clear. They have mentioned in first contribution.*

A: We appreciate your insightful suggestions. We agree that it is better to provide contribution of the study. We discussed them at the end of the introduction by paragraph and bullet.

2. *Several paragraphs contain trivial information and should be dropped.*

A: Thank you very much. We've rewritten them.

3. *Each section should have a summary table. If contents are too much, then add summary tables for the subsection.*

A: Thank you very much. We summarized the related work section in Table 1 of the revision.

4. *Each section should present new information and perspective to enlighten the readers.*

A: We appreciate your insightful suggestions. We've rewritten them.

5. *Paper contributions are presented without pitching problems in recent studies. Add one paragraph before it to highlight issues in recent studies and at the end how this paper overcome those shortcomings.*

A: We appreciate your insightful suggestions. We showed the gap by summarizing related works with table.

6. *Improve the presentation and resolution of table 1. It’s a very informative figure.*

A: We appreciate your insightful suggestions. We updated it as Table 2

7. *Figures should be improved as it’s a very important figure that shows the reduction scheme*

A: We appreciate your insightful suggestions. We updated it as Figure # 2, 3 and 4

8. *Writing is good, need to check the typo errors.*

A: Thank you very much. We were able to get away with it thanks to linguistics

9. *paper is well-formatted, plz check the formatting of the reference*

A: Thank you very much. We have checked as much as possible.

10. *equations should be explained well and checked again*

A: Thank you very much. We have checked and modified equation # 2,3 in the revsion

11. *I found some English mistakes please check them.*

A: Thank you very much. We were able to get away with it thanks to linguistics

**Reviewer 4**

1. *List out the main contributions of the proposed work.*

A: We appreciate your insightful suggestions. We agree that it is better to provide contribution of the study. We discussed them at the end of the introduction by paragraph and bullet.

2. *The related works can be summarized as a table.*

A: Thank you very much. We summarized the related work section in Table 1 of the revision.

3. *Some of the recent works such as the following can be discussed in the paper "An ensemble machine learning approach through effective feature extraction to classify fake news, Variance ranking attributes selection techniques for binary classification problem in imbalance data".*

A: Thank you very much. We have site this paper [15] in the revision.

4. *Compare the current work with recent state-of-the-art.*

A: Thank you very much. We have compared the result as shown in figure 4 and Table 7 of the revision.

5. *Present a detailed analysis on the results obtained.*

A: We appreciate your insightful suggestions. We have rewritten the result and discussion section

6. *The English language used in the paper can be improved.*

A: Thank you very much. We were able to get away with it thanks to linguistics.

Thanks very much to editor and reviewers for your precious suggestions！
